# Applying Research to Public Health Questions: Biologically Relevant Exposures

**DOI:** 10.1289/ehp.1002015

**Published:** 2010-04

**Authors:** Linda S. Birnbaum

**Affiliations:** National Institutes of Health, Department of Health and Human Services, Research Triangle Park, North Carolina, E-mail: birnbaumls@niehs.nih.gov

Complex diseases have both genetic and environmental components. Understanding the contribution of environmental factors to disease susceptibility will require a more comprehensive view of exposure and biological response than has traditionally been applied.

“Exposure” is defined as the “contact between an agent and a target” (World Health Organization 2004). For risk assessment, this definition of “exposure” has been applied primarily to the individual or human population as a target of exposure, and to a chemical as an agent of exposure; however, the target of exposure can be an organ, tissue, or cell, and the agent of exposure can be a biological, physical, or psychosocial stressor or the by-product of a given exposure agent. Exposure science is required to incorporate consideration of lifestage, genetic susceptibility, and interaction of nonchemical stressors for holistic assessment of risk factors associated with complex environmental disease. Achieving this goal will require the establishment of new capabilities to identify biologically relevant exposure metrics that can be directly associated with key events in a disease process and with an individual’s exposure profile.

Wild (2005) proposed the need for a “step change” in exposure assessment and articulated a vision for exposure measurement calling for an “exposome,” or measurement of the life-course of environmental exposures to provide the evidence base for public health decisions to address environmental health. Wild and others (e.g., Weis et al. 2005) discussed the potential of emerging technologies to provide this new generation of exposure information. In their guest editorial in *EHP*, Smith and Rappaport (2009) argued that if we expect to have any success at identifying the contribution of environmental factors on chronic diseases, “we must develop 21st-century tools to measure exposure levels in human populations” and quantify the exposome. The National Academy of Sciences committee on Emerging Science for Environmental Health Decisions, sponsored by the National Institute of Environmental Health Sciences (NIEHS), organized a workshop in February 2010 that launched a discussion on resources needed to make the exposome a reality.

The Exposure Biology Program of the National Institutes of Health Genes, Environment and Health Initiative, led by the NIEHS, invests in innovative new technologies to determine how environmental exposures—including diet, physical activity, stress, and drug use—contribute to human disease. These technologies include sensors for chemicals in the environment, and new ways to characterize dietary intake, levels of physical activity, responses to psychosocial stress, and measures of the biological response to these factors at the physiologic and molecular levels. These new tests will provide the improved accuracy and precision needed to determine how environmental and lifestyle factors interact with genetic factors to determine the risk of developing disease.

As changes in the field of exposure rapidly accelerate, The International Society of Exposure Science (ISES) and the *Journal of Exposure Science and Environmental Epidemiology* (*JESEE*) have partnered on a project marking the 20th anniversary of the formal establishment of ISES (Bahadori and Barr 2010). Each *JESEE* issue in 2010 will contain one or two mini-reviews that showcase successes in exposure science that have had a broad impact on understanding exposures, improving public health, and impacting policy. Although the focus of these reviews will be on celebrating historical successes, they will also highlight the relevance to some of the most pressing public health issues we face today. The final *JESEE* issue of 2010 will feature future scientific directions that are expected to have a profound impact on the field of exposure science and related disciplines. This initiative dovetails with the activities of the National Research Council as they begin work on a major new study cosponsored by the U.S. Environmental Protection Agency and the NIEHS, “Human and Environmental Exposure Science in the 21st Century.”

Understanding the connection between our health and our environment, with its mixture of chemicals, diet, and lifestyle stressors, is no less complex than understanding the intricacies of the human genome. Here at the NIEHS, we remain committed to helping the field of exposure science evolve to meet emerging public health challenges. We look forward to the increased contributions of exposure scientists as we work to understand to role of environment in etiology of disease.

## Figures and Tables

**Figure:**
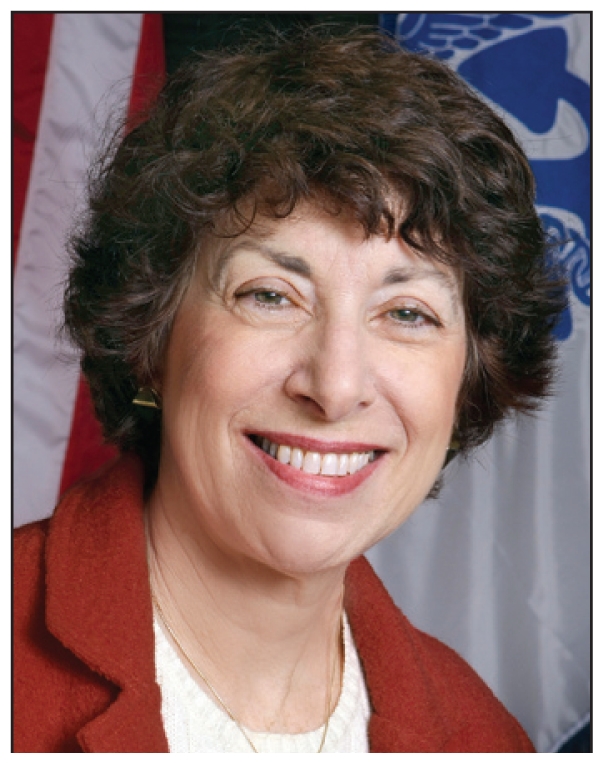
Linda S. Birnbaum
